# Supplementary material for: KLF4, Slug and EMT in Head and Neck Squamous Cell Carcinoma
Source: Cells. 2021 Mar 3;10(3):539. doi: 10.3390/cells10030539 (PMC7998447; doi:10.3390/cells10030539)
Supplement: Supplementary file 1 [file cells-10-00539-s001.pdf]

Supplementary Material

Supplementary Table S1.

List of used PCR primers in the Study.

Gene	Forward primer	Reverse primer	Reference
GAPDH	TGCACCACCAACTGCTTAGC	GGCATGGACTGTGGTCATGAG	[1]
ZEB1	CATTTTTCCTGAGGCACCTG	TGAAAATGCATCTGGTGTTCC	[1]
ZEB2	GGGACAGATCAGCACCAAAT	CGCAGGTGTTCTTTCAGATG	[1]
TWIST	AGAAGTCTGCGGGCTGTG	CGTCTGCAGCTCCTCGTAAG	[1]
SNAIL	CCTCCCTGTCAGATGAGGAC	GCCTCCAAGGAAGAGACTGA	[1]
KLF4	TCCAAAGAAGAAGGATCTCGGCCA	AACGTGGAGAAAGATGGGAGCA	[1]
SNAI2	ACATAAGCAGCTGCACTGCG	ATGGGTCTGCAGATGAGCCC	[2]

References:

1. Li, X.L.; Hara, T.; Choi, Y.; Subramanian, M.; Francis, P.; Bilke, S.; Walker, R.L.; Pineda, M.; Zhu, Y.; Yang, Y., et al. A p21-ZEB1 complex inhibits epithelial-mesenchymal transition through the microRNA 183-96-182 cluster. *Mol Cell Biol* **2014**, *34*, 533-550, doi:10.1128/MCB.01043-13.
2. Du, B.; Shim, J.S. Targeting Epithelial-Mesenchymal Transition (EMT) to Overcome Drug Resistance in Cancer. *Molecules* **2016**, *21*, doi:10.3390/molecules21070965.

Supplementary Technical Supporting Data-1

Further input on experiments to differentiate between Slug and Snail, and other technical data.

Experiments to differentiate between Slug and Snail in HNSCC tissue samples

Interestingly, immunohistochemical reactions with Slug antibodies produced for the whole recombinant protein, as most of the commercial ones including the clone S43-1259 (BD Pharmingen), hardly distinguish between Snail and Slug proteins (**Supplementary Figure 1**). Even western blot does not allow a distinguish between Snail and Slug, because both proteins show a 30 kDa product. Only few amino acids, short peptide components allow a differentiation between Snail and Slug (**Supplementary Figure 1**), but antibodies even used in papers, which distinguish between Snail and Slug [25] are frequently withdrawn by providers. This is an important problem, while since the publication of Ye et al. [7], it is highly important to distinguish between Snail and Slug.

In addition, the mouse monoclonal Snail antibody (clone G-7, Santa Cruz Biotechnology, Heidelberg, Germany), which recognizes the peptide sequence exclusive in Snail and not present in Slug protein (**Supplementary Figure 1**), does not show positive reaction in HNSCC tissue, which is intensive stained by Slug antibody (not shown).

Supplementary Figure S1

Commercial antibodies against Snail or Slug do not allow the distinguish between the two proteins

Slug protein sequence

(Source: NCBI, Protein Blast:

National Center for Biotechnology Information, U.S. National Library of Medicine 8600 Rockville Pike, Bethesda MD, 20894 USA)

MPRSFLVKKHFNASKKPNYSELDTHTVIISPYLYESYSMPVIPQPEILSSGAYSPITVWTTAAPFHAQLP
NGLSPLSGYSSSLGRVSPPPPSDTSSKDHSGSESPISDEEERLQSKLSDPHAIEAEKFQC NLCNKTYSTF
SGLAKHKQLHCDAQSRKSFSCKYCDKEYVSLGALKMHIRTHTLPCVCKICGKAFSRPWLLQGHIRT
HTGEKPFSCPHCNRAFADRSNLRAHLQTHSDVKKYQCKNCSKTFSRMSLLHKHEESGCCVAH

If whole recombinant protein was used for producing SLUG antibody, as in the case of commercial antibodies the following coverage is found for SNAI2 gene product: SLUG protein:

Range 1: 1 to 268GenPeptGraphics Next Match Previous Match

Alignment statistics for match #1

Score	Expect	Method	Identities	Positives	Gaps
553 bits(1426)	0.0	Compositional matrix adjust.	268/268(100%)	268/268(100%)	0/268(0%)

```
Query  1    MPRSFLVKKHFNASKKPNYSELDTHTVIISPYLYESYSMPVIPQPEILSSGAYSPITVWT
60
            MPRSFLVKKHFNASKKPNYSELDTHTVIISPYLYESYSMPVIPQPEILSSGAYSPITVWT
Sbjct  1    MPRSFLVKKHFNASKKPNYSELDTHTVIISPYLYESYSMPVIPQPEILSSGAYSPITVWT
60

Query  61   TAAPFHAQLPNGLSPLSGYSSSLGRVSPPPPSDTSSKDHSGSESPISDEEERLQSKLSDP
120
            TAAPFHAQLPNGLSPLSGYSSSLGRVSPPPPSDTSSKDHSGSESPISDEEERLQSKLSDP
Sbjct  61   TAAPFHAQLPNGLSPLSGYSSSLGRVSPPPPSDTSSKDHSGSESPISDEEERLQSKLSDP
120

Query  121  HAIEAEKFQCNLCNKTYSTFSGLAKHKQLHCDAQSRKSFSCKYCDKEYVSLGALKMHIRT
180
            HAIEAEKFQCNLCNKTYSTFSGLAKHKQLHCDAQSRKSFSCKYCDKEYVSLGALKMHIRT
Sbjct  121  HAIEAEKFQCNLCNKTYSTFSGLAKHKQLHCDAQSRKSFSCKYCDKEYVSLGALKMHIRT
180

Query  181  HTLPCVCKICGKAFSRPWLLQGHIRTHTGEKPFSCPHCNRAFADRSNLRAHLQTHSDVKK
240
            HTLPCVCKICGKAFSRPWLLQGHIRTHTGEKPFSCPHCNRAFADRSNLRAHLQTHSDVKK
Sbjct  181  HTLPCVCKICGKAFSRPWLLQGHIRTHTGEKPFSCPHCNRAFADRSNLRAHLQTHSDVKK
240

Query  241  YQCKNCSKTFSRMSLLHKHEESGCCVAH  268
            YQCKNCSKTFSRMSLLHKHEESGCCVAH
Sbjct  241  YQCKNCSKTFSRMSLLHKHEESGCCVAH  268
```

If whole recombinant protein was used for producing SLUG antibody, as in the case of commercial antibodies, the following coverage is found for SNAI1 gene product: SNAIL protein:

```
Query  1    MPRSFLVKKHFNASKKPNYSEL---DTHTVIISPYLYESYSMPVIPQPEILSSGAYSPIT
57
            MPRSFLV+K  + ++KPNYSEL   +       PY  +++ +  IP PEIL+  A  P+
Sbjct  1    MPRSFLVRKPSDPNRKPNYSELQDSNPEFTFQQPY-DQAHLLAAIPPPEILNPTASLPML
59

Query  58   VWTTAAPFHAQLPNGLSPLSGYSSSLGRVSPPPPSDTSSKDHS---GSESPISDEEERLQ
114
            +W +     AQ      P++  S  L + SP     TS  D     GS+ P
Sbjct  60   IWDSVLAPQAQ------PIAWASLRL-QESPRVAELTSLSDEDSGKGSQPPSPPSPAPSS
112

Query  115  SKLSDPHAIEAEKFQCNLCNKTYSTFSGLAK-HKQLHC-----DAQSRKSFSCKYCDKEY
168
               +   ++EAE          Y+ F GL +  KQL       D Q+RK+F+CKYC+KEY
Sbjct  113  FSSTSVSSLEAE---------AYAAFPGLGQVPKQLAQLSEAKDLQARKAFNCKYCNKEY
163

Query  169  VSLGALKMHIRTHTLPCVCKICGKAFSRPWLLQGHIRTHTGEKPFSCPHCNRAFADRSNL
228
            +SLGALKMHIR+HTLPCVC  CGKAFSRPWLLQGH+RTHTGEKPFSCPHC+RAFADRSNL
Sbjct  164  LSLGALKMHIRSHTLPCVCGTCGKAFSRPWLLQGHVRTHTGEKPFSCPHCSRAFADRSNL
223

Query  229  RAHLQTHSDVKKYQCKNCSKTFSRMSLLHKHEESGC  264
            RAHLQTHSDVKKYQC+ C++TFSRMSLLHKH+ESGC
Sbjct  224  RAHLQTHSDVKKYQCQACARTFSRMSLLHKHQESGC  259
```

As seen here, there is a high range overlap between snail and slug at protein level.

The peptide sequence of SNAIL amino acids 113-139 is not present in Slug.

FSSTSVSSLEAEAYAAFPGLGQVPKQL

The antibody produced against Snail amino acids 113-139 has full reactivity with Snail; BUT NO REACTION WITH SLUG AT ALL.

This antibody is available for Santa Cruz Biotech and can be found by the following link:

https://datasheets.scbt.com/sc-271977.pdf

In our material, tissue samples showing high reaction with Slug antibodies do not react with this Santa Cruz Biotech antibody.

Supplementary Technical Supporting Data-2

Gene expression analysis of SNAI1, SLUG, ZEB1, TWIST and KLF4 in HNSCC compared with normal oral mucosa

The CT-values of SNAI1 ranged from 34 to 38 both in control normal mucosa, and in HNSCC. Dissociation curve analysis and sequencing did not validate a specific product for SNAI1 in the used tissue samples.

The CT-values of ZEB1 ranged from 31 to 34 in normal mucosa, and from 30 to 35 in HNSCC. The CT-values of TWIST ranged from 35 to 37 in normal mucosa, and from 32 to 37 in HNSCC. ZEB1 showed low expression, but its sequence was validated. All PCR products were also amplified and subjected to agarose gel electrophoresis. The electrophoresis image of all PCR products displayed single bands, which were the same size as the one published in the original publications of the primers (**Supplementary Table 1**).

Supplementary Figure S2.

The % of positive cells and staining intensity of Slug is higher in cases with increased SNAI2 gene expression.

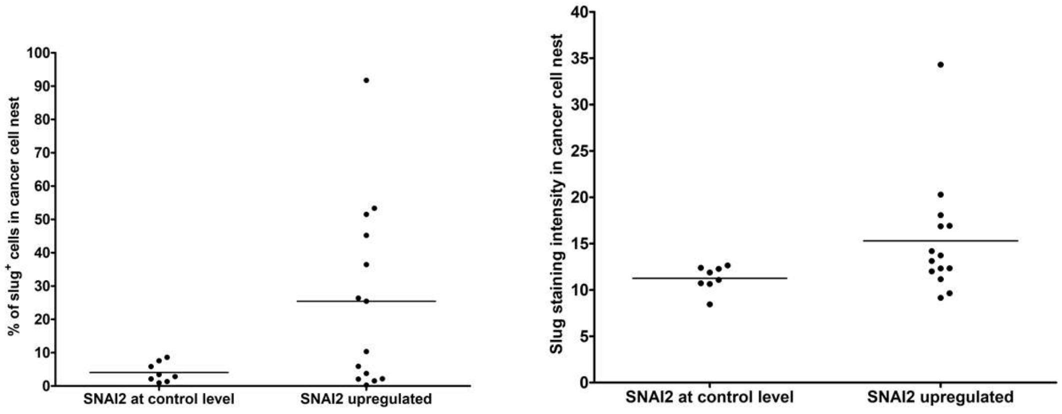

Although, a significant correlation between immunohistochemical Slug staining intensity or the frequency of the stained cells among tumor cells and SNAI gene expression was not possible to state, both the % of Slug positive cells in cancer cell nests as well as the Slug staining intensity were higher in HNSCC cases with upregulated SNAI2 gene expression than in the cases with SNAI2 gene expression level at the normal control mucosa.

Supplementary Figure S3.

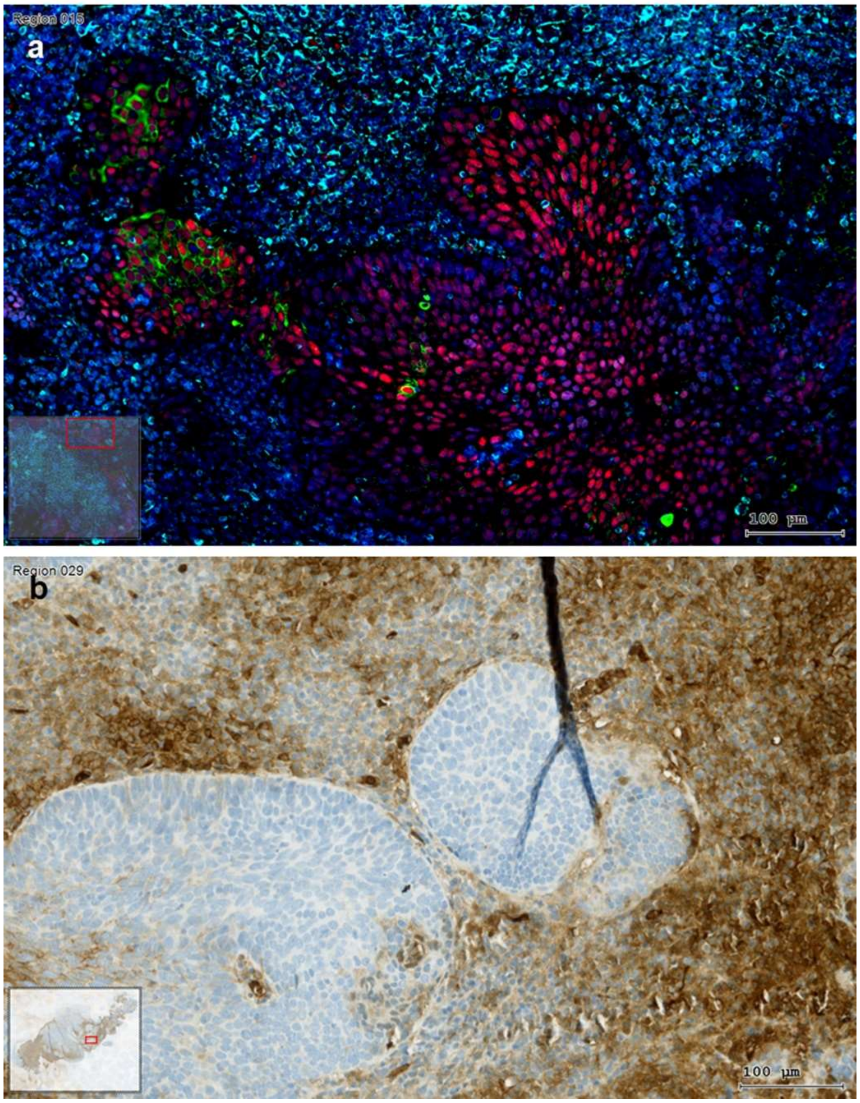

Comparison of immunofluorescence labeling of KLF4 (red), pan-cytokeratin (green) and vimentin (light blue) combined (a) with enzyme immunohistochemical labeling of TGF-β1 (b) in HPV^{+} HNSCC with wild type p53. In the cancer cell nests the tumor cell nuclei contained intensive KLF4-reaction, CK co-localized with KLF4 in numerous cells, but it did not co-localize with vimentin. In vimentin^{+} stoma cells of (**a**) intensive TGF-β1 staining was detected (**b**)**.** Bars: a-b: 100 µm.
